# Putative Causal Variant on *Vlgr1* for the Epileptic Phenotype in the Model Wistar Audiogenic Rat

**DOI:** 10.3389/fneur.2021.647859

**Published:** 2021-06-09

**Authors:** Samara Damasceno, Pablo Augusto de Souza Fonseca, Izinara Cruz Rosse, Márcio Flávio Dutra Moraes, José Antônio Cortes de Oliveira, Norberto Garcia-Cairasco, Ana Lúcia Brunialti Godard

**Affiliations:** ^1^Departamento de Genética, Ecologia e Evolução, Instituto de Ciências Biológicas, Universidade Federal de Minas Gerais, Belo Horizonte, Brazil; ^2^Departamento de Farmácia, Escola de Farmácia, Universidade Federal de Ouro Preto, Ouro Preto, Brazil; ^3^Departamento de Fisiologia e Biofísica, Instituto de Ciências Biológicas, Universidade Federal de Minas Gerais, Belo Horizonte, Brazil; ^4^Departamento de Fisiologia, Faculdade de Medicina de Ribeirão Preto, Universidade de São Paulo, Ribeirão Preto, Brazil

**Keywords:** WAR, audiogenic model, seizure predisposition, mutation, sequencing, *Vlgr1*, molecular marker

## Abstract

Wistar Audiogenic Rat is an epilepsy model whose animals are predisposed to develop seizures induced by acoustic stimulation. This model was developed by selective reproduction and presents a consistent genetic profile due to the several generations of inbreeding. In this study, we performed an analysis of WAR RNA-Seq data, aiming identified at genetic variants that may be involved in the epileptic phenotype. Seventeen thousand eighty-five predicted variants were identified as unique to the WAR model, of which 15,915 variants are SNPs and 1,170 INDELs. We filter the predicted variants by pre-established criteria and selected five for validation by Sanger sequencing. The genetic variant c.14198T>C in the *Vlgr1* gene was confirmed in the WAR model. *Vlgr1* encodes an adhesion receptor that is involved in the myelination process, in the development of stereocilia of the inner ear, and was already associated with the audiogenic seizures presented by the mice Frings. The transcriptional quantification of *Vlgr1* revealed the downregulation this gene in the corpus quadrigeminum of WAR, and the protein modeling predicted that the mutated residue alters the structure of a domain of the VLGR1 receptor. We believe that *Vlgr1* gene may be related to the predisposition of WAR to seizures and suggest the mutation *Vlgr1*/Q4695R as putative causal variant, and the first molecular marker of the WAR strain.

## Introduction

Epilepsy is a chronic neurological condition that affects people of all ages and an estimated over 2 million people diagnosed per year (1). Different factors may act in the pathogenesis of this disorder, including genetic and molecular alterations. Gitaí and collaborators (2008) suggested that genes may be involved in epilepsy as the primary cause of the condition, determining the threshold of susceptibility or in response to epileptic insults (2). The association of genes to epilepsy has important implications for research and clinical practice. Once, the identification of candidate genes may direct the studies and helps in the understanding of the biological processes involved in the susceptibility and expression of seizures, which allows the development of treatments directed to specific mechanisms. In the clinical context, the genetic test is the most important potential application, either to clarify the diagnosis in patients or to predict the occurrence of epilepsy in people with a family history (3).

Animal models are indispensable for elucidating the genetic and molecular aspects of epilepsy and many have been developed in an attempt to mimic human epilepsy characteristics. The Wistar Audiogenic Rat (WAR) strain is a reflex epilepsy model developed by selective reproduction of Wistar rats sensitive to acoustic stimuli (4). When exposed to a high-intensity sound, these animals exhibit generalized tonic-clonic seizures similar to the seizures presented in humans. In WAR these are named audiogenic seizures, involve auditory pathway structures and are dependent on the brainstem circuitry (4–6). Through repetitive stimulation, forebrain structures are recruited and the seizures phenotype is modified to a limbic seizures pattern in a process called kindling (7). These aspects make WAR a versatile model for the study of ictogenic processes, which may contribute to the study of reflex, tonic-clonic and limbic seizures.

Currently, the WAR strain is maintained in two separate colonies more than 25 years old, one located at the Faculdade de Medicina de Ribeirão Preto da Universidade de São Paulo (FMRP-USP) where the strain was developed, and another located at the Instituto de Ciências Biológicas da Universidade Federal de Minas Gerais (ICB-UFMG). In both colonies, the animals presents a consistent genetic profile due to the homozygosity established during more than 50 generations of inbreeding (8). Although the model is well-characterized as regards the behavioral, electrophysiological and cellular aspects, the studies aiming at genetic and molecular alterations related to susceptibility to seizures and their consequences are still scarce (8, 9).

Some studies have demonstrated alterations of gene expression in WAR that had seizures (9–12), and recently our research group published the differential transcriptome of the WAR model by RNA sequencing (RNA-Seq) using samples of the corpus quadrigeminum of WAR and Wistar rats (13). After comparing the transcriptional profiles, we identified differentially regulated genes in the WAR strain and demonstrated that there are genes with differential expression inherent to the model, regardless of the occurrence of seizures and that the seizures can alter gene regulation (13). In addition to the observed molecular alterations, we believe that the predisposition of the WAR model to audiogenic seizures may be related to the fixation of some alleles due to inbreeding. Based on this hypothesis, the present study was performed in order to identify genetic variants through the analysis of WAR and Wistar RNA-Seq data. This analysis is able to provide information about mutations and molecular targets that may be contributing to the epileptic phenotype.

## Materials and Methods

### Animals

Some of the samples used in this study were obtained during the study of Damasceno and collaborators (13). Sixteen WAR and sixteen Wistar 70 days' old male rats were provided by the Animal Facility of the University Federal de Minas Gerais (UFMG). The animals were housed in groups of three in the same cage and kept in temperature controlled (22 ± 2°C) under a 12/12 h light–dark cycle with food and water available *ad libitum*. These rats were divided into four groups of eight animals each: Control naïve and WAR naive with animals not subjected to acoustic stimulation. Control stimulated and WAR stimulated with animals subjected to the acoustic stimulation protocol (11, 14, 15). During stimulation, animals were evaluated by the severity index (SI) (4, 16). The Control stimulated group was composed of Wistar rats that did not respond to sound and Wistar rats that responded to sound with wild running and jumping (SI ≤ 0.23). The WAR stimulated group was composed of WARs that presented generalized tonic-clonic seizures and clonic spasms (SI ≥ 0.85). At PND-80 (96 h after the stimulation) all the animals were euthanized and had their brains dissected.

The other part of the samples evaluated in this study were obtained from animals provided by the Animal Facility of the University of São Paulo (USP). Fourteen WAR and fourteen Wistar 70 days' old male rats, not subjected to acoustic stimulation (naïve) and kept under the same conditions, were euthanized, their brains dissected, and DNA samples donated to UFMG researchers. Measures were applied to minimize the pain and discomfort of the animals and all animal experimentation was carried out in compliance with institutional guidelines and approved by the Ethics Committee for Animal Use of UFMG (CEUA-UFMG), protocol number 251/2012 and by the Ethics Committee for Animal Use of USP (CEUA-USP), protocol number 181/2016.

### Data Analysis

**Figure 1:**
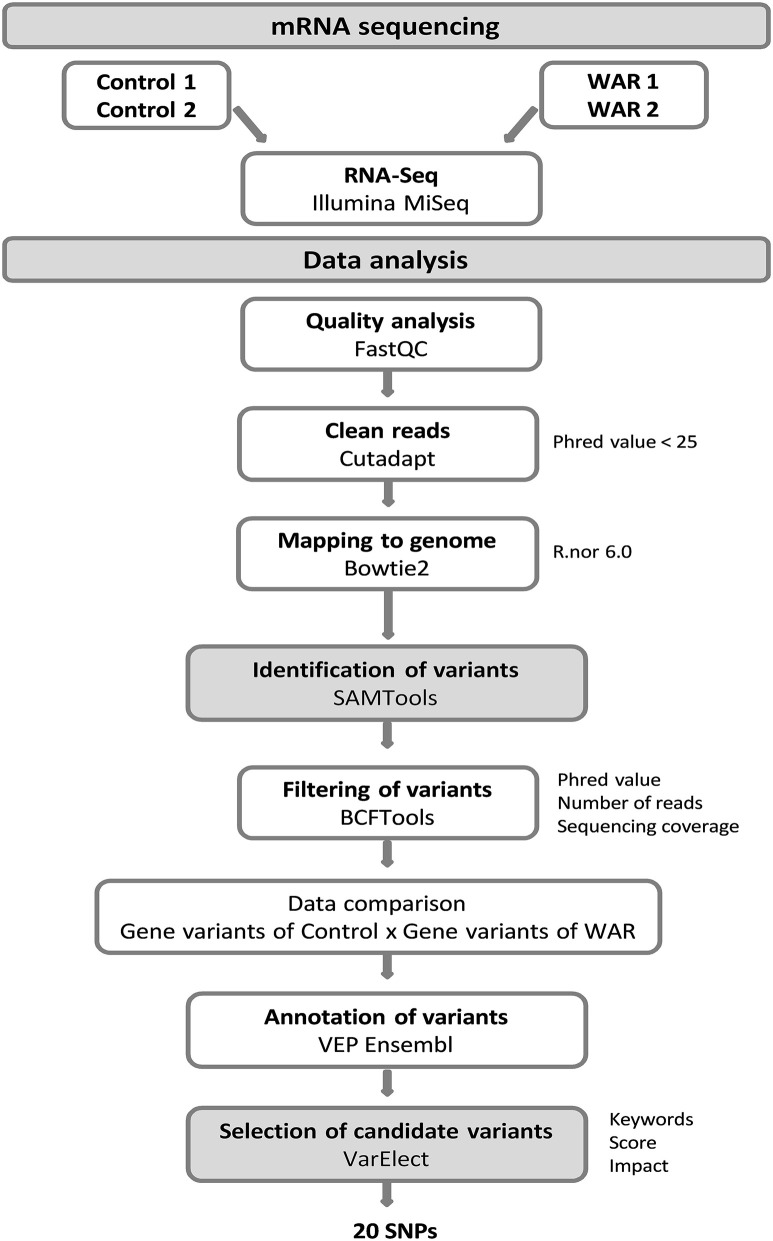
Flowchart of data analysis for the variant's identification. The mRNA sequencing was performed with two WAR and two controls rats. The raw data were evaluated by a quality control tool and submitted to trimming, which Phred value < 25 was used to trim the reads. The filtered reads were mapped in the reference genome. The identification of genetic variants was performed by the software SAMTools and filtered by quality criteria using BCFTools. The sample data were grouped and compared Control vs. WAR. The exclusive predict variants of each group were annotated using VEP Ensembl. Gene prioritization analysis based in keywords was performed using software VarElect and other criteria, such as score values and impact, were used for the selection of candidate variants.

The RNA-Seq data were obtained during the work performed by Damasceno and collaborators (13) (Accession: GSE152339 https://www.ncbi.nlm.nih.gov/geo/). The raw data generated by the RNA-Seq were initially evaluated by the FastQC quality control tool and trimmed by the Cutadapt software, which Phred value <25 was used to trim the reads. The filtered reads were mapped based on the reference genome R.nor 6.0 (*Rattus novergicus*) using the Bowtie2 software. The identification of genetic variants was performed by the SAMTools software. In this step, SNP (Single Nucleotide Polymorphisms) and INDEL (Insertion and Deletion) variants were identified for each sequenced sample (Control 1; Control 2; WAR 1; WAR 2) in relation to the reference genome. Then the variants were filtered by quality criteria using BCFTools software. The criteria were Phred value >20, minimum of two reads representing the less frequent allele and minimum coverage of four reads for the region, criteria adapted from Rosse and collaborators (17). The data of the samples were merged according to the groups, Control and WAR, the genetic variants were compared between them and the exclusive variants of each group were annotated using VEP (Variant Effect Predictor) software (Figure 1).

### Candidate Variants Selection

Following the annotation, the genes list with the exclusive variants of the WAR was submitted to functional data-mining by VarElect software. This uses information contained in the GeneCards database and performs a gene prioritization analysis based on the relationship of select keywords and phenotype. In order to filter the genes with the exclusive variants of the WAR group we used the following set of words: “epilepsy;” “seizure;” “kindling;” “audiogenic seizure;” “brainstem;” “auditory pathway.” Subsequently, the list of prioritized genes generated by VarElect was screened considering only variants in genes directly related to the phenotype with a score greater than the mean of all scores plus twice the standard deviation [Score > $\bar{x}$ + (2 × SD)]. Due to the large number of variants filtered, we applied an additional selection criterion, considering as candidates only the predicted variants with moderate and high functional impact (Figure 1).

### Primer Design

Gene sequences were obtained from the Ensembl Genome Browser database (http://www.ensembl.org/index.html) and all primer pairs were designed using the Primer3 software (http://bioinfo.ut.ee/primer3-0.4.0/primer3/). The primers for the genotyping were designed to amplify up to 500 base pairs (bp), being 250 bp upstream and downstream of the genetic variant. The primers to evaluate the amount of transcripts were designed to align in different exons with amplicon of up to 200 bp. The quality and specificity of the primers were evaluated using the software NetPrimer (http://www.premierbiosoft.com/netprimer/), and Primer-BLAST (https://www.ncbi.nlm.nih.gov/tools/primer-blast/), respectively. Primers were synthesized by IDT–Integrated DNA Technologies (Síntese Biotecnologia) (Table 1).

**Table 1:** Sequences of the primers designed for genotyping and quantitative PCR.

**Gene symbol**	**Forward (5^**′**^**−**3^**′**^)**	**Reverse (5^**′**^**−**3^**′**^)**	**Product size (bp)**	**Annealing Tm (^**°**^C)**
*Chrna4*	TTATCCGATGAACAGGTCCAGA	CTGTGGTCATCTGAGCTTAAACCT	379 (gDNA)	59.5
*Grin2a*	TTTCTGAGAGTCGGCGATGTAT	ATCTCCAGGCATGTAAGTCTCCT	406 (gDNA)	59.9
*Grin2b*	ACATCTGATAAAGCTCCCATTGAT	GAATTGTTTTCACCCTTGCTCA	437 (gDNA)	57.8
*Kcnq3*	GCGACCAAGAATCACTACCTGT	TGTCACAATGACCGACCTGTAC	432 (gDNA)	60.3
*Vlgr1*	TGAGGTTCTGCCCAATCAGTA	GCATAGGAAAGGGGTCACTGT	457 (gDNA)	59.2
Vlgr1^*^	CAATCTTGCCTCACACCATACA	CTTTCAATCCTTTGGCTTTCC	133 (cDNA)	57.1
Gdi2^**^	CCCGCCATGAATGAGGAAT	TCACTGACATTATCCCCGACA	70 (cDNA)	59.3
Rps26^**^	CGTGCTTCCCAAGCTCTATGT	CGATTCCTGACAACCTTGCTATG	75 (cDNA)	60.0

* *Primers used in transcript analysis,*

** *reference gene*.

### Genotyping

The genotyping by Sanger sequencing was performed for the validation of five predicted variants. DNA samples from 16 Wistar and 16 WAR from the UFMG were initially sequenced. These samples were obtained from the same animals used by Damasceno and collaborators (2018). Subsequently, the genotyping was performed using DNA samples from 14 Wistar and 14 WAR from USP, obtained by donation. DNA extraction was performed according to the protocol of DNeasy Blood & Tissue Kit (Qiagen). The resuspended DNA was quantified using the Qubit 2.0 Fluorometer (Invitrogen) and the samples presented 260/230 nm and 260/280 nm ≥ 1.8. The genetic material of each animal was subjected to conventional PCR using the enzyme DreamTaq DNA Polymerase (Thermo Fisher Scientific) and the primers specific for each selected variant. The PCR products were purified using polyethylene glycol 8000 (20% PEG / 2.5 M NaCl) and the samples prepared in microtubes: 20–30 ng of purified DNA, 1 uL Primer forward (10 uM), and DNAse and RNAse free-water for the final volume of 7.5 uL. The Sanger sequencing was performed by Myleus Facility (Myleus Biotechnology).

The data obtained were submitted to quality control, which Phred value lower than 20 was used to trim the reads and the sequences were evaluated using the CodonCode Aligner software (CodonCode Corporation) where the electropherograms were analyzed for the detection of the variants. The predicted variants from the RNA-sequencing data were considered validated when identified also by Sanger sequencing in the WAR samples. Subsequently, the allele and genotype frequencies were calculated and submitted to statistical analysis.

### Quantitative PCR

The relative quantification of *Vlgr1* transcripts was performed with corpus quadrigeminum cDNA samples from eight WAR and eight Wistar rats from UFMG. These samples were obtained from animals in the groups Wistar naive (Wis-N) and WAR naive (WAR-N) not subjected to acoustic stimulation, as described by Damasceno and collaborators (2018). In order not to create bias, samples from animals subjected to acoustic stimulation were not used. The reactions were performed using the CFX 96 TM Real Time system (BioRad) and the protocol of the intercalating agent SYBR Fast qPCR Kit Master Mix (Kapa Biosystems). The housekeeping genes *Gdi2* (GDP dissociation inhibitor 2) and *Rps26* (Ribosomal protein S26) were used as reference gene to normalize mRNA levels. Samples were analyzed in duplicates, with each reaction containing: 10 μL of SYBR Fast qPCR Master Mix; 1 μL of cDNA (10 ng); 0.8 μL of each primer (forward and reverse, 10 μM); and 8.2 μL DNAse and RNAse-free water. The cycling conditions were according to the protocol of the intercalating agent. In all reactions a negative control was tested and added melting curves that were analyzed to assure the absence of spurious products. The obtained data were evaluated according to Vandesompele and collaborators (18).

### Statistical Analysis

With the allele and genotype frequencies, the chi-square test (X^2^) was performed to test the Hardy-Weinberg equilibrium. The Fisher exact test was performed to test the hypothesis of association between the genotype and the epileptic phenotype in the WAR model. The relative quantification data of the *Vlgr1* gene were analyzed for normality distribution using the D'Agostino-Pearson and Shapiro-Wilk tests. The relative mRNA of the groups was evaluated using unpaired *t*-test. Analyses were performed using GraphPad Prism and results were considered statistically significant at *p* ≤ 0.05.

### Protein Modeling

The gene whose variant was validated, *Vlgr1*, was studied using databases. This gene encodes the 6298-residue protein (Uniprot: A0A096MK89), which is composed of multiple domains for which no experimental structure is currently available. We modeled the Calx-beta domain where the mutation is located (residues 4,649–4,730) using threading as implemented in I-Tasser (19). The best model has an estimated RMSD (Root Mean Square Deviation) of 2.7 ± 2 Å. Conservation and coevolution analysis was performed using the PFstats software (20). A multiple sequence alignment for the Calx-beta domain was obtained from Pfam (21). In order to remove fragments and redundancy, only sequences with at least 80% of the HMM (Hidden Markov Models) size was kept in the alignment, and the alignment was also filtered by 70% identity.

## Results

### Identification and Selection of Candidate Variants

**Figure 2:**
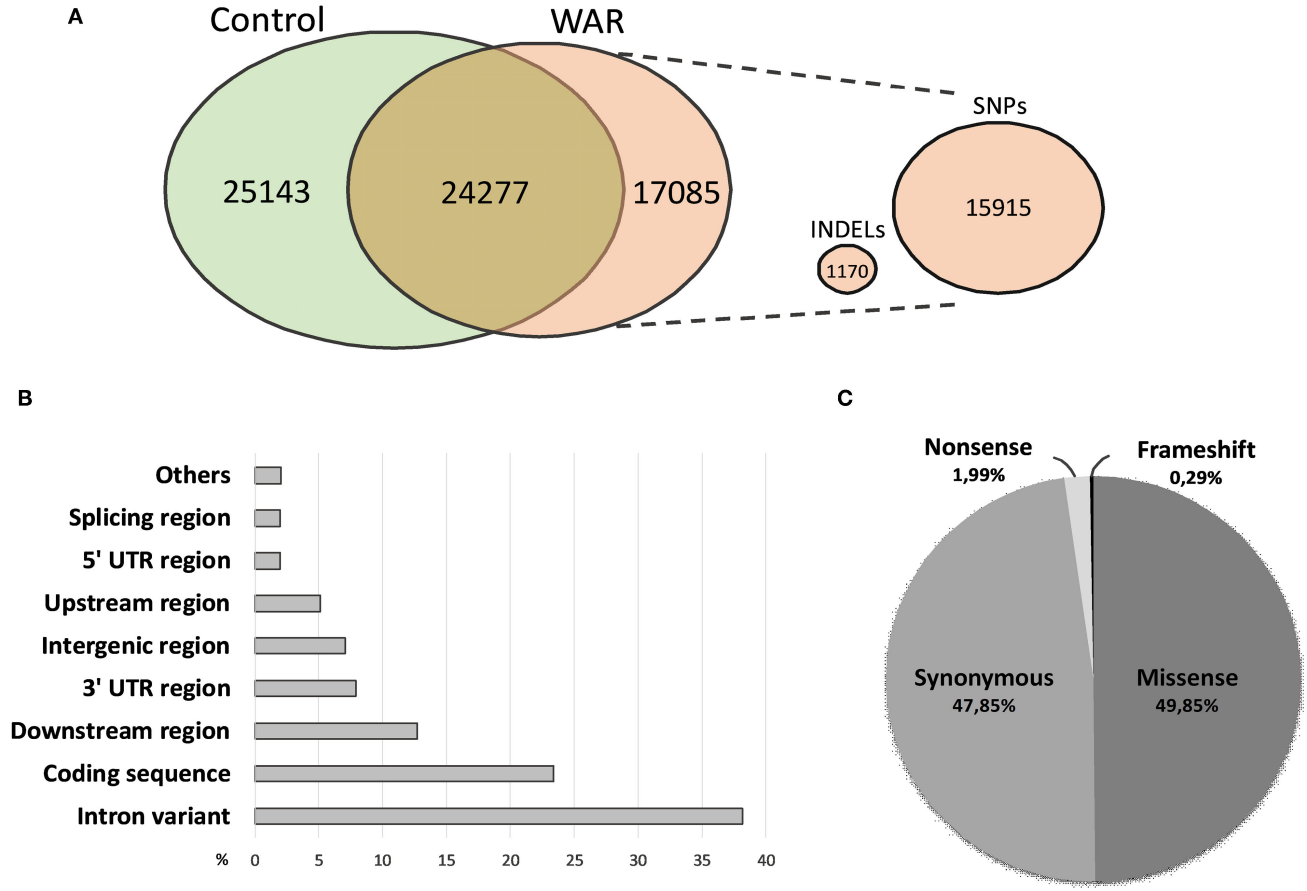
Number of genetic variants identified and functional annotation. **(A)** Venn diagram of the number of variants in the groups evaluated and number of INDELs and SNPs exclusive of the WAR model. **(B)** Annotation of the exclusive variants of the WAR model according to the location in the gene. **(C)** Annotation of the variants localized in coding sequence according to the functional consequence.

Sequence data from Wistar and WAR allowed us to identify genetic variants SNPs (Single Nucleotide Polymorphisms) and INDELs (Insertion and Deletion). For each sample, more than forty million predicted variants were identified and about 60% were filtered out based on the criteria of Phred value, number of reads and minimum coverage (Table 2). In the WAR model, 17,085 predicted variants were identified as unique to the model, with 15,915 variants SNPs and 1,170 INDELs (Figure 2A). The functional annotation performed with the 17,085 genetic variants revealed that more than 23% are mapped in coding sequences (Figure 2B). Most of these variants are missense or synonyms and, in minority, nonsense or frameshift (Figure 2C).

**Table 2:** Variant's data.

**Library**	**Variants identified**	**Variants filtered (%)**
Control 1	42,235	25810 (61.11)
Control 2	63,836	41574 (65.13)
WAR 1	40,914	23440 (57.29)
WAR 2	60,003	36730 (61.21)

*Number of predicted variants identified in the sequenced libraries and the number of variants filtered based on the criteria of Phred value, number of reads and minimum coverage*.

For the selection of candidate variants, the genes harboring predicted variants exclusive to the WAR model were subjected to a functional prioritization analysis and, 55 genes (harboring 162 SNPs and 9 INDELs) were prioritized. Subsequently, these variants were filtered based on the predicted functional impact resulting in 20 candidate SNP distributed in 14 genes. These variants presented 6.2 X mean depth coverage, with a minimum of 4.0 X and a maximum of 9.0 X (Table 3). Five variants were selected for validation by Sanger sequencing: the missense variants in the *Chrna4, Grin2a, Grin2b* and *Vlgr1* genes, and the nonsense variant in the *Kcnq3* gene. These five variants were selected considering the literature, in search of some an already described association of these genes with different types of epilepsy and, unpublished data with audiogenic models, developed by our group and collaborators. Most of these genes encode ion-channel-coupled receptors. *Chrna4* encodes a subunit of the nicotinic acetylcholine receptor whose mutations are associated with nocturnal frontal lobe epilepsy (22, 23). *Grin2a* and *Grin2b* encodes subunits of the glutamate ionotropic receptor NMDA and their mutations are related to idiopathic focal epilepsy and epileptic encephalopathy, respectively (24, 25). *Kcnq3* encodes a subunit of the voltage-gated potassium channel whose mutations are associated with benign familial neonatal seizures (26). *Vlgr1* encodes a receptor of the G protein-coupled adhesion receptor family, also named Mass1 (Monogenic, Audiogenic Seizure Susceptibility), this gene is associated with audiogenic epilepsy in the model Frings mice and related to the development of stereocilia in hair cells of the inner ear (27, 28).

**Table 3:** Genetic variants candidates and annotation details.

**Gene symbol**	**Chromossome**	**Variant Location**	**Consequence**	**Impact**	**Depth** **coverage**	**Allele**	**Codons**	**Amino** **acids**	**cDNA** **position**	**CDS** **position**	**Protein** **position**
Chrna4^*^	3q43	NC_005102.4:176546328	missense	moderate	8	G	gTg/gCg	V/A	465	233	78
*Col4a1*	16q12.5	NC_005115.4:83606642	missense	moderate	7	A	Ccc/Acc	P/T	2,276	2,074	692
*Depdc5*	14q21	NC_005113.4:83180280	missense	moderate	6	C	gaT/gaG	D/E	1,388	1,170	390
*Depdc5*	14q21	NC_005113.4:83186890	missense	moderate	8	G	tTt/tCt	F/S	880	662	221
*Foxp2*	4q21	NC_005103.4:41877682	missense	moderate	7	C	Tgt/Cgt	C/R^(rs64524464)^	1,331	697	233
Grin2a^*^	10q11	NC_005109.4:6027269	missense	moderate	8	T	Ccc/Tcc	P/S	914	772	258
Grin2b^*^	4q43	NC_005103.4:169609308	missense	moderate	6	C	gAc/gGc	D/G	2,058	1,268	423
Kcnq3^*^	7q34	NC_005106.4:106752718	nonsense	high	5	T	tgT/tgA	C/-	657	657	219
*Kcnq3*	7q34	NC_005106.4:106742931	missense	moderate	6	C	aaC/aaG	N/K	1,284	1,284	428
*Kcnq3*	7q34	NC_005106.4:106746213	missense	moderate	5	T	Ggc/Agc	G/S	1,135	1,135	379
*Mtor*	5q36	NC_005104.4:165280946	missense	moderate	5	A	gaT/gaA	D/E	2,069	2,004	668
*Mtor*	5q36	NC_005104.4:165353292	missense	moderate	7	G	Acc/Gcc	T/A	5,646	5,581	1,861
*Nipa1*	1q22	NC_005100.4:114422581	missense	moderate	4	C	agC/agG	S/R	150	150	50
*Otof*	6q14	NC_005105.4:27400991	missense	moderate	4	C	Gag/Cag	E/Q	1,289	1,180	394
*Polg*	1q31	NC_005100.4:141183524	missense	moderate	8	G	Tgg/Cgg	W/R	1,081	883	295
*Reln*	4q11	NC_005103.4:9670839	missense	moderate	8	G	Agc/Ggc	S/G	3,448	3,448	1,150
*Reln*	4q11	NC_005103.4:9675405	missense	moderate	9	A	gTc/gAc	V/D	3,698	3,698	1,233
*Reln*	4q11	NC_005103.4:9748380	missense	moderate	5	G	aAa/aGa	K/R	8,849	8,849	2,950
*Tcf4*	18q12.1	NC_005117.4:65388874	missense	moderate	4	A	atG/atA	M/I	1,049	495	165
Vlgr1^*^	2q11	NC_005101.4:9309379	missense	moderate	4	C	cAa/cGa	Q/R	14,198	14,084	4,695

*Genes, location, and consequences of variants.*

* *Genetic variants selected for genotyping by Sanger sequencing*.

### Genotyping and Statistical Evaluation

**Figure 3:**
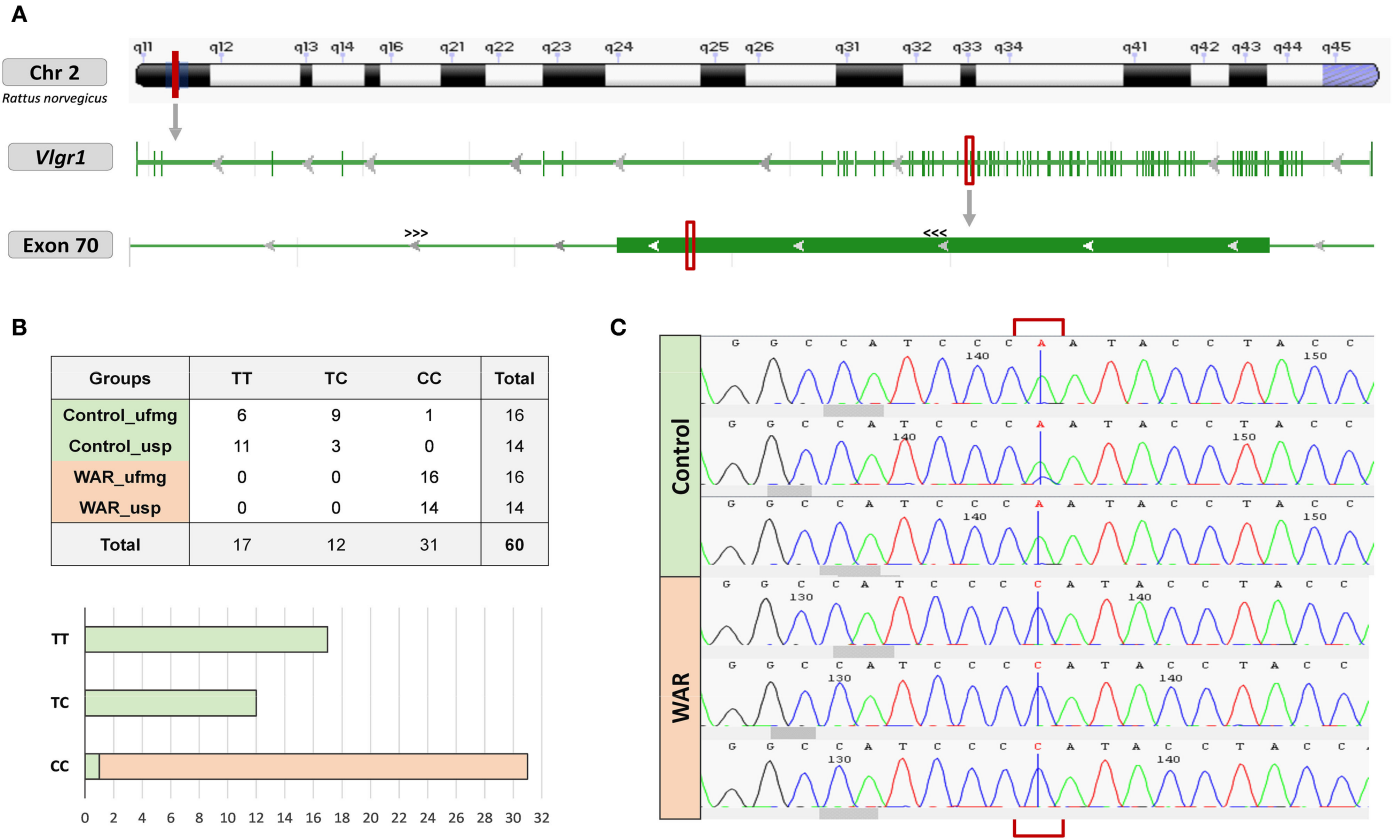
Localization and genotyping of the predicted variant c.14198T> C in the *Vlgr1* gene. **(A)** Location of the variant at chromosome, gene and exon level. The annealing positions of primers used in genotyping are indicated by ^>>><<<.^ **(B)** Genotypes of the control animals and WAR from different colonies. All genotyped WAR from UFMG and USP are homozygous for the mutated allele while the controls animals are heterozygous or homozygous for the wild-type allele. **(C)** Electropherograms of genotyping by Sanger sequencing. Representation of two homozygous controls for the wild-type allele, one heterozygous control and three homozygous WAR for the mutated allele.

The variants in the *Chrna4, Grin2a, Grin2b, Kcnq3* and *Vlgr1* genes were initially sequenced in animals from UFMG (16 Wistar and 16 WAR) e posteriorly in animals from USP (14 Wistar and 14 WAR). The predicted variant c.14198T>C in the *Vlgr1* gene was confirmed in both datasets. The other variants evaluated were not confirmed, as the alternative allele was not identified in the Sanger sequencing. The genotyped WARs were homozygous for the mutant allele of the *Vlgr1* gene, consequently fixed in the WAR animals from UFMG. The *Vlgr1* gene is located on chromosome 2 and the missense variant was identified in the final portion of exon 70 (Figure 3A). With the sequencing of animals from USP we were able to exclude the possibility that the variant confirmed in the *Vlgr1* gene is a de novo mutation, a hypothesis raised due to the long period of isolation between the colonies. All genotyped WAR animals from USP were also homozygous for the mutant allele, being c.14198T>C in the Vlgr1 gene fixed in both colonies of the WAR model (UFMG and USP). Considering the evaluated animals of both colonies, 100% of the WAR were homozygous for the C allele presenting CC genotype and the controls are heterozygous or homozygous for the T wild-type allele presenting TC or TT genotypes with the exception of a single Wistar individual which presented CC genotype as the WAR model (Figures 3B,C). Based on the observed genotypes, we found a significant difference between the genotype frequencies of WAR and controls animals indicating that the variant in the *Vlgr1* gene is not in Hardy-Weinberg equilibrium ($X_{1}^{2}$ = 56.13; *p* ≤ 0.05). Furthermore, when testing the hypothesis of association between the genotype and the epileptic phenotype in the WAR model, we observed a significant association (Fisher's exact test; *p* ≤ 0.05).

### Quantitative PCR and Protein Modeling

**Figure 4:**
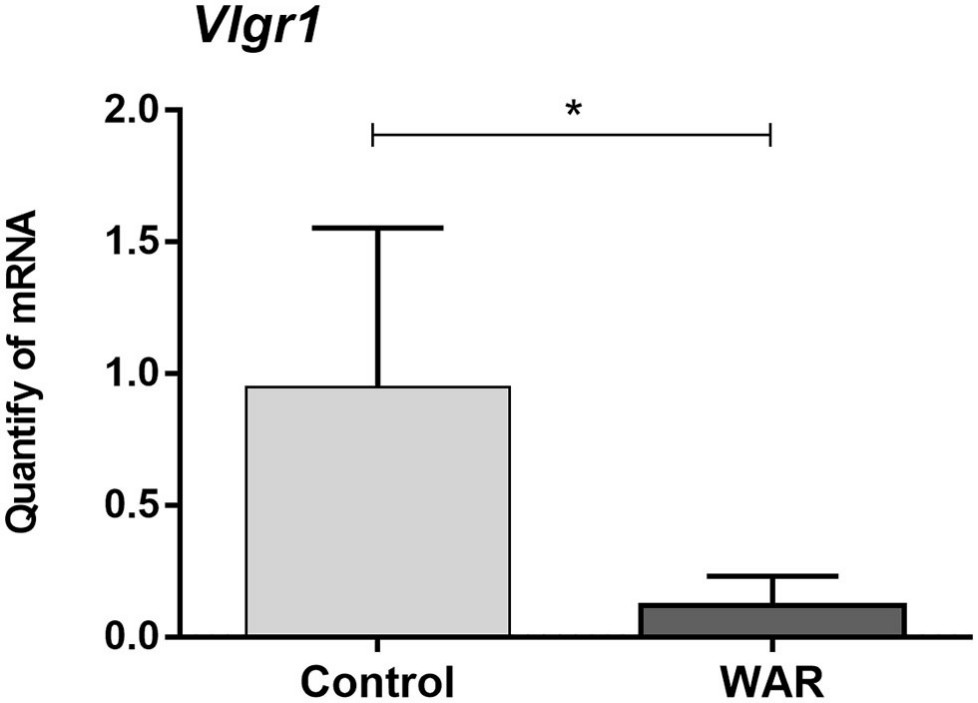
Relative quantification of *Vlgr1* gene transcripts in the corpus quadrigeminum. X-axis: Relative quantities of mRNA in arbitrary units. Y-axis: Experimental groups: naïve Wistar rats (Control) and naive WAR (WAR). Bars represent mean ± SEM. Statistical analysis: T Student test, *p ≤ 0.05.

**Figure 5:**
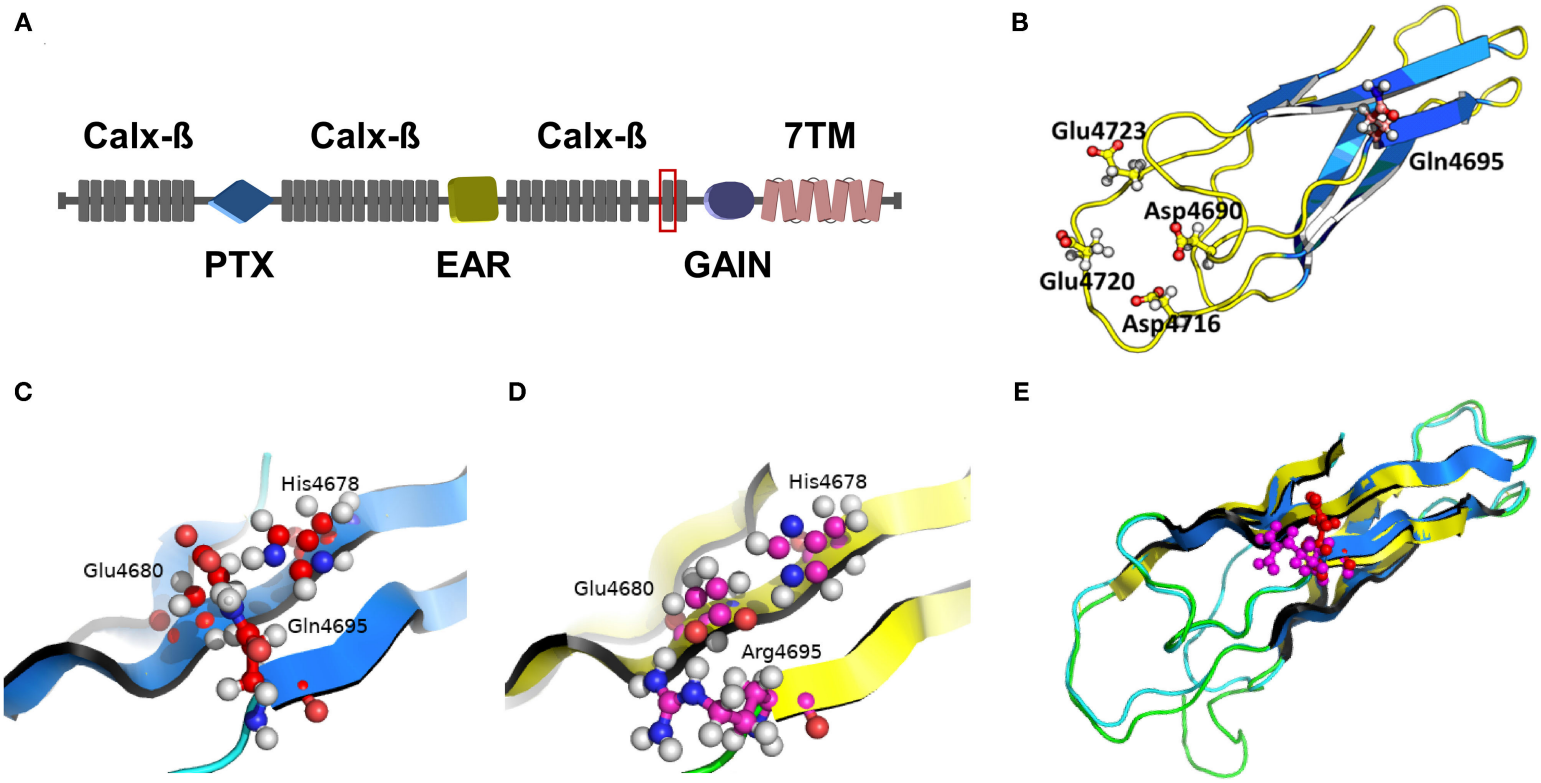
VLGR1 receptor architecture and three-dimensional structure of the mutated domain. **(A)** Basic architecture of the VLGR1 receptor with the different domains and mutated site location. Calx-β (Calcium Exchanger β); PTX (Pentraxin); EAR (Epilepsy Associated Repeat); GAIN (autoproteolysis-inducing GPCR); 7 TMB (Seven-transmembrane). **(B)** Structure of the wild-type Calx-β domain (residues 4,649–4,730) showing the mutation site (Gln4695) and four highly conserved residues (Asp4690, Asp4716, Glu4720 and Glu4723). **(C)** Larger image of the wild-type Calx-β domain showing Gln4695 and, Glu4680 and His4678 residues that form a saline bridge on the neighboring beta-sheet. **(D)** Larger image of the mutated Calx-β domain showing the change of amino acid Gln to Arg in residue 4,695 and its interaction with neighboring beta-sheet preventing saline bridge formation. **(E)** Overlap of modeled Calx-β domains (wild type and mutated). Wild-type domain: Gln4695 residue in red, beta sheet in blue and other structures in light blue; Mutate domain: Arg4695 residue in pink; beta-sheet in yellow and other structures in light green. In this image we observe a change in the structure of the mutated domain near the position of conserved residues Asp4716 and Asp4690 (light green color).

The relative quantification of transcript levels of the *Vlgr1* gene (*t* = 3.451; *p* ≤ 0.05) revealed that this gene down-regulated in the corpus quadrigeminum of naive WAR animals when compared to naive controls (Figure 4). The *Vlgr1* gene encodes the Very Large G protein-coupled Receptor 1 (VLGR1) that has different domains: PTX domain (Pentraxin); EAR / EPTP domain (Epilepsy Associated Repeat/Epitempin Repeat); Calx-β domain (Calcium Exchanger β); GAIN domain (GPCR Autoproteolysis-inducing); 7TM domain (Seven-transmembrane). The missense variant c.14198T>C confirmed in the WAR model corresponds to one of the Calx-β repeats (Figure 5A). At protein level this mutation results in the change of residue 4,695 from an amino acid glutamine to arginine (p.Gln4695Arg or p.Q4695R). Protein modeling of the wild-type Calx-β domain (residues 4,649–4,730) revealed that the structure consists of two parallel beta-sheets with four highly conserved residues (Asp4690, Asp4716, Glu4720 and Glu4723) (Figure 5B). The residue Gln4695 is present in one of the beta sheets, next to residues Glu4680 and His4678 that form the only saline bridge in the neighboring beta-sheet (Figure 5C). The domain structure showed that the formation of this bridge depends on the frequency of specifically charged near residues. While the individual rate of positive and negative residues for all Calx-β domains in the equivalent positions of His4678 and Glu4680 is are 12.3 and 9.7%, respectively, the presence of an oppositely charged residue in the other positions raise those rates to 20.5 and 16.2%, respectively. The p.Q4695R mutation results in the change from an amino acid with neutral electric charge to a positively charged amino acid. The protein modeling predicts that the Arg4695 residue interferes with the structure of the neighboring beta-sheet, preventing the formation of the saline bridge (Figure 5D). Moreover, through the overlap of the models (wild-type and mutant domain) we observed a change in the domain structure near the Asp4716 and Asp4690 conserved residues (Figure 5E).

## Discussion

The increase of homozygosity is one of the consequences of generations of inbreeding as it occurred in the development of the WAR strain and still occurs in the maintenance of colonies (8). Among the variants selected for genotyping in the present study one was confirmed, c.14198T>C in the *Vlgr1* gene. This variant was validated in WAR animals from different colonies (UFMG and USP) indicating that the allele was fixed during the strain selection process. The *Vlgr1* gene encodes a receptor of the G protein-coupled adhesion receptor family (aGPCR) that has already been related to the process of myelination in the central nervous system (CNS), stereocilia organization of inner ear hair cells, febrile seizures and audiogenic seizures (29–32).

The VLGR1 receptor, the largest known cell surface protein, is highly expressed in the embryonic nervous system, which suggests a role in CNS development (33). VLGR1 has been reported to regulate myelin-associated glycoprotein (MAG) expression in response to extracellular calcium (32, 34). The VLGR1 is highly expressed in oligodendrocytes and myelinated regions of superior and inferior colliculi of the adult mice (32). The suppression of VLGR1 signaling reduces up-regulated of MAG and the expression of this protein is reduced in the brain of the audiogenic epilepsy model, Frings mice (*Vlgr1* mutant), suggesting that the myelination of these mice may be modified by the absence of the receptor and be a contributor to epileptic seizures (32). In the present work, we observed that *Vlgr1* gene is down-regulated in the corpus quadrigeminum of WAR. We do not have data about the myelination of this structure, and we cannot directly associate the down-regulation of this gene with the mutation, but we can speculate that the signaling via VLGR1 may be impaired, allowing possible consequences in the process of myelination of the WAR model.

Frings mice are a model of audiogenic epilepsy resulting from a nonsense mutation in the *Vlgr1* gene that results in a truncated protein (29). The deletion of a nucleotide at exon 31, a region corresponding to one of the Calx-β repeats between the PTX domain and the EAR domain, results in the premature termination of the encoded protein (*Vlgr1*/V2250X) (27, 35). In addition to the Frings mice, there are three other models that show mutation in the *Vlgr1* gene. BUB/BnJ mice that have a mutation identical to the Frings model (*Vlgr1*/V2250X) (26), the knockin mice model (*Vlgr1*/del7TM) that does not express the transmembrane and cytoplasmic domains of VLGR1 (34), and the knockout mice model (*Vlgr1*-/-) that were developed by deletion directed from exon 2 to 4 of the *Vlgr1* gene (36). All these models, like the Frings, are susceptible to audiogenic seizures, but the underlying mechanisms that relate VLGR1 to the occurrence of these seizures are still unknown (26, 34, 35). One of the hypotheses is based on the involvement of VLGR1 in CNS myelination. However, VLGR1 is also involved in the development of hair bundles of sensorial cells of the inner ear (28, 30, 37).

The hair bundles are composed of numerous stereocilia that are arrayed in rows and interconnected by different links that maintain their integrity and stability: tip link, top connectors, shaft connectors and ankle link (30). VLGR1 acts on the basal part of the stereocilia forming the ankle links, transitory links that aid in the structuring of the hair bundles and are lost around the second postnatal week (28, 30, 38). In the mutant models for the *Vlgr1* gene, the development of the stereocilia seems compromised (30, 37, 39). In *Vlgr1*-/- mice and *Vlgr1*/del7TM it was observed that the ankle links are not formed and the hair bundles become disorganized and inclined just after birth (30, 37). Other components of the ankle links were not found in the region of the link in the *Vlgr1*-/- mice, suggesting that the formation of this complex is dependent on the presence of the functional VLGR1 (28, 40). These models indicate an important role of this receptor in the development of the auditory system and in the transduction of sound information (40). Scanning electron microscopy data revealed alterations in the hair bundles of the internal and external sensory cells in the WAR model (41). Interruptions were observed in the arrangement of stereocilia in the row of inner hair cells and irregularity in the hair bundles of the outer hair cells, being concentrically closed and exhibiting heterogeneity in the size of the stereocilia (41). Although we do not have data for VLGR1 expression in the cochlear structure of WAR, we hypothesize that the abnormalities observed in this structure may be a consequence of changes in expression and/or functionality of this receptor possibly influenced by the mutation.

The *Vlgr1* gene has been related to epilepsy in different contexts. In humans, a nonsense mutation in the EAR domain of this gene (*Vlgr1*/S2652X) was identified in a family with febrile seizures (31) and, *Vlgr1* gene expression reduction was observed in patients with low-grade glioma who presented epileptic seizures as a symptom. It is suggested that the low expression of *Vlgr1* is a risk factor and can be considered as a marker for patients with glioma at risk of epileptic seizures (42). In the WAR model we confirmed a missense mutation in this gene and its down-regulation in the corpus quadrigeminum. The differential quantification of transcripts suggests a reduced expression of the VLGR1 receptor in this structure that is essential for the initiation and propagation of epileptiform activity. However, this point requires further investigation with the use of specific antibodies, for example, which was not possible during the performance of this study due to the failure of the different antibodies tested. In collaboration with other research groups, strategies are being evaluated to overcome this limitation and move forward with the study of the variant.

The mutation *Vlgr1*/Q4695R occurs in a region corresponding to one of the Calx-β domain repeats where the residue Gln4695 in the wild-type protein is located near to two residues (His4678 and Glu4680) that form a saline bridge in the neighboring beta sheet. By protein modeling we observed that the mutation results in the absence this saline bridge and consequent alteration in the domain structure. This may be due to the difference in charge between the residues since the formation of this bridge depends on the frequency of specifically charged near residues. Possibly the change from Gln4596 with neutral electric charge to Arg4695 with positive electric charge altered the stability of the interactions. This may be due to the favored interaction between the Arg4695 and Glu4680 which prevents Glu4680 residue from binding with the His4678 residue for the formation of saline bridge.

The predicted structural changes for the mutated domain allow us to suggest an impairment in VLGR1 receptor functionality in WAR model. Thus, a possible impairment in VLGR1 receptor signaling could be predisposing these animals to seizures by different processes such as, changes in CNS development, neuronal myelinization and steriocilia development. In the present work, it was not possible to measure the damage caused by the *Vlgr1*/Q4695R mutation and to elucidate a specific mechanism by which the receptor may be favoring the development of seizures. However, considering the data and bibliography presented we believe that the *Vlgr1* gene may be related to the predisposition of WAR animals to seizures and suggest the mutation *Vlgr1*/Q4695R as putative causal variant, and the first molecular marker of the WAR strain. This is the first work performed with the WAR model in search of genetic variants and the data presented here may direct different research aiming to elucidate the altered mechanisms that make WAR and other models susceptible to audiogenic seizures.

## Data Availability Statement

The dataset analyzed in this study can be found in NCBI GEO under the accession number GSE152339 (https://www.ncbi.nlm.nih.gov/geo/).

## Ethics Statement

The animal study was reviewed and approved by Ethics Committee for Animal Use of UFMG (CEUA-UFMG), protocol number 251/2012 and by the Ethics Committee for Animal Use of USP (CEUA-USP), protocol number 181/2016.

## Author Contributions

SD conducted all the experiments and wrote the paper. PASF assisted in the identification analysis of genetic variants. ICR assisted in protein modeling. MFDM provided WAR rats from the breeding colony of UFMG. NG-C and JACO provided DNA samples from WAR and Wistar rats of the USP. ALBG supervised all the study and corrected the manuscript. All authors contributed to the article and approved the submitted version.

## Conflict of Interest

The authors declare that the research was conducted in the absence of any commercial or financial relationships that could be construed as a potential conflict of interest.
